# Numerical Investigation on the Thermodynamic Characteristics of a Liquid Film upon Spray Cooling Using an Air-Blast Atomization Nozzle

**DOI:** 10.3390/e22030308

**Published:** 2020-03-09

**Authors:** Jia-Xin Li, Yun-Ze Li, En-Hui Li, Tong Li

**Affiliations:** 1 School of Aeronautic Science and Engineering, Beihang University, Beijing 100191, China; jxin.lee@buaa.edu.cn (J.-X.L.); lienhui@buaa.edu.cn (E.-H.L.); 2 Institute of Engineering Thermophysics, North China University of Water Resources and Electric Power, Zhengzhou 450045, Henan, China; 3 Advanced Research Center of Thermal and New Energy Technologies, Xingtai Polytechnic College, Xingtai 054035, Hebei, China; 4 Chengyi Academy of PKUHS, Peking Univeristy, Beijing 100080, China; litong@i.pkuschool.edu.cn

**Keywords:** air-blast spray cooling, thermodynamic characteristic, discrete phase model method, liquid film, spray droplet

## Abstract

This paper developed a three-dimensional model to simulate the process of atomization and liquid film formation during the air-blast spray cooling technological process. The model was solved using the discrete phase model method. Several factors including the thermodynamic characteristics of the liquid film as well as the spray quality with different spray mass flow rates under different spray heights were numerically investigated and discussed. The results show that the varied spray height has little effect on the Sauter Mean Diameter (d_32_) of the spray droplet, while the thermodynamic characteristics of liquid film including the liquid film height, the liquid film velocity, and the liquid film generation rate are sensitive to the change of the spray height. With the growth of spray mass flow rates, d_32_, the liquid film generation rate and liquid film height become larger, while the liquid film velocity with different spray mass flow rates has a similar velocity distribution, indicating that the spray mass flow rate has little effect on the liquid film velocity. The average d_32_ of droplet size shows a sharp drop when sprayed from the nozzle in a short period of time (<1.5 ms), then approaching smoothness, below a value of 40 μm, the spray status tends to be stable.

## 1. Introduction

During the last several decades, the miniaturization, as well as the highly integrated, high- density device packaging, and inefficiency of electronics have evoked great interest in modern industry and a wide variety of aids from industrial and environmental fields have been applied [1,2], for instance, ground-based devices like the lateral diffused metal oxide semiconductor field effect transistor (LD-MOSFET) [3] and space devices like the NASA Goddard mars orbiter laser altimeter [4]. However, large amounts of waste heat generated at high heating power, cause a super high temperature so as to decrease the service lifetime and efficiency of the electronic devices. Therefore, a high-efficiency thermal management approach, whose heat dissipation requirement would be over an order of 10^2^ W/cm^2^ [5], is increasingly needed to remove the waste heat due to the dramatic growth in both electrical power and local heat flux of the electronics devices.

Tremendous studies [5,6,7] regarding the heat transfer attainability and discovering that spray cooling has long enjoyed a reputation as a cooling method being one of the most effective methods for removing high heat-waste flux. It has been proved that its utility in dissipating high heat fluxes could be up to 1200 W/cm^2^ using water as the working fluid [7]. Besides, spray cooling has several other remarkable advantages including its latent heat of vaporization which absorbs a great amount of heat with a minimal temperature variation and thermal uniformity with low coolant mass flux in a given condition. The spray cooling phenomenon can be described as follows: working fluid is forced through the atomization nozzle where the liquid is broken into numerous tiny droplets due to the pressure. Subsequently, droplets impinge onto a heated surface, forming a thin liquid film upon the surface and taking away heat. The waste heat can be dissipated by the heat transfer mechanism of forced convection, film evaporation, nucleate boiling, and second nucleation [8,9].

For enhancing the cooling capacity of spray cooling, numerous researches regarding the complicated heat transfer mechanisms have been carried out through both numerical simulation investigations and experimental studies under different conditions. In the existing literature, it has been found that droplet-impacting and the thin liquid film are the critical factors affecting the performance of the spray cooling system when using the same coolant. In general, a better spray cooling performance can be acquired by decreasing the Sauter Mean Diameter (SMD) of the spray droplet size [10] and increasing the velocity of droplet-impacting [11]. It has been found that the twin- fluid atomization method can obtain a higher quality of spray, and the air-blast nozzle is one representative example [12,13,14]. On the other side, the cooling ability of the spray shows that the heat transfer characteristics are influenced significantly by low gravity, which may result in a lower cooling effectiveness than higher gravity and normal gravity due to the increasing thickness of the average liquid film [15].

Experimental research is the major method to investigate the heat transfer performance of spray cooling technology. However, the conclusions achieved by experimental conditions have been usually case- specific and could not obtain a mechanical arrangement. Spray cooling involves many complicated processes, such as liquid atomization, droplet interaction, and liquid film formation, thus it is very difficult to complete research of the entire mechanism and model the full process. Therefore, many researchers have attempted to build a model to investigate the mechanism of the spray cooling technology for achieving an ideal heat transfer capacity. Some researchers set up a three-dimensional model to investigate the heat transfer mechanism of spray cooling technology. In these studies, a multiphase flow model was used to simulate the surface temperature, the liquid film thickness, and the film velocity distribution [16,17,18,19,20]. The two-dimensional model also has been established for modelling a single droplet impacting on a flat surface with a constant surface temperature [2,21,22,23]. The volume of fluid method was used in these researches. The droplet diameter was 50–150 μm and the droplet velocity was 1.0–10.0 m/s. The simulation results indicated that the decrease of the droplet diameter and the increase of the velocity can promote the performance of spray cooling technology. Some researchers studied the cooling performance of spray cooling in harmful environments [24,25], such as the over-load environment.

Looking through the previous researches, most of the investigations have focused on the average liquid film thickness, not on the thermodynamic characteristics of the liquid film. In the present study, the Commercial CFD software ANSYS (15.0) (ICEM CFD and FLUENT) was used to carry out the numerical investigation with the discrete phase model (DPM) method, simulating the process of atomization and liquid film formation and summarizing the SMD of the droplet size and the thermodynamic characteristics. This included the distribution of liquid film height (LFH), liquid film velocity (LFV), and liquid film generation rate (LFGR) when using the air-blast nozzle under different spray height (SH) and varied spray mass flow rates (SMFR).

## 2. Computational Model and Numerical Analysis

### 2.1. Physical Model and Its Finite Element Mesh Generation

**Figure 1:**
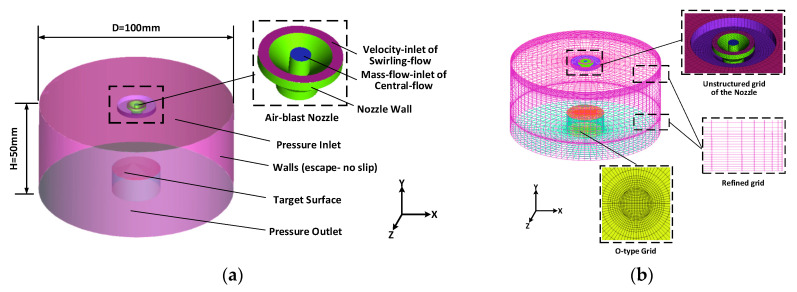
Schematic view, (**a**) 3-D physical model and (**b**) mesh.

A 3-D CFD model of a cylindrical region equivalent to the working spray chamber with a diameter of D (100 mm) and a height of H (50 mm) had been established by the commercial 3D modeling software SOLIDWORKS (2017), which schematic view is shown in Figure 1 and the detailed structure of the air-blast nozzle is also displayed. Since the FLUENT’s air-blast atomizer model does not include the internal gas flows, the inlets of air streams were created on the top the model including the mass-flow-inlet of central-flow, velocity-inlet of swirling-flow and pressure- inlet. The bottom is for the outlet of pressure and the other surface of the cylindrical region is for the numerical escape walls with no slip. The target surface on the top of the internal cylinder, has height and area of 8 mm and 1.131 cm^2^ (diameter, d = 1.2 cm) respectively.

Figure 1b demonstrates the mesh generation of the computational model. The structured hexahedron grid combined with the unstructured tetrahedron grid were applied to generate the finite element meshes of the model. Additionally, the advanced O-type grid was used to promote the quality of mesh. In order to improve the accuracy of calculation and simulation results, mesh densification was carried out for the jet center area and the target surface region. The solution domain was divided into $2.5\times 10^{6}$ meshes and this grid refinement could be shown to lead to grid independence solutions.

### 2.2. Governing Equations and Boundary Conditions

In the present study, the Commercial CFD software ANSYS (15.0) (ICEM CFD and FLUENT) was used to carry out the finite element numerical investigation. A Discrete-Phase particle trajectory model based on the Euler–Lagrangian method was adopted, in which the gas-phase is regarded as a continuous phase, and the Euler method was used to solve the Navier–Stokes conservation equations for the continuous phase; the particle phase is treated as the discrete phase, and the particle trajectory is solved by the Lagrangian method. Except for the heat and mass transfer caused by particles, it is assumed that the movements of droplet particles along their respective orbits do not interfere with each other, and there is no turbulent diffusion of droplet particles.

#### 2.2.1. Continuous Phase Model

The continuous phase is the air, and the mass equation, momentum equation, and energy equation can be described as Equations (1) to (3) respectively [2]:

(1) $$\frac{\partial \rho }{\partial t}+\frac{\partial }{\partial x}\left(\rho u_{x}\right)+\frac{\partial }{\partial y}\left(\rho u_{y}\right)+\frac{\partial }{\partial z}\left(\rho u_{z}\right)=0$$

(2) $$\left[\begin{array}{l} \frac{\partial \left(\rho u_{x}\right)}{\partial t}+\nabla \cdot \left(\rho u_{x}U\right)=\nabla \cdot \left(\mu \nabla u_{x}\right)-\frac{\partial \rho }{\partial x}+F_{x}\\ \frac{\partial \left(\rho u_{y}\right)}{\partial t}+\nabla \cdot \left(\rho u_{y}U\right)=\nabla \cdot \left(\mu \nabla u_{y}\right)-\frac{\partial \rho }{\partial y}+F_{y}\\ \frac{\partial \left(\rho u_{z}\right)}{\partial t}+\nabla \cdot \left(\rho u_{z}U\right)=\nabla \cdot \left(\mu \nabla u_{z}\right)-\frac{\partial \rho }{\partial z}+F_{z} \end{array}\right]$$

(3) $$\frac{\partial \left(\rho E\right)}{\partial t}+\nabla \cdot \left[U\left(\rho E+p\right)\right]=\nabla \cdot \left[\lambda_{eff}\nabla T-\sum_{j}J_{j}\int_{T_{ref}}^{T}c_{p,j}dT+\left(\tau_{eff}\cdot U\right)\right]+S_{h}$$

where E is the specific energy including kinetic energy, intrinsic energy, and potential energy; $\lambda_{eff}$ is the effective thermal conductivity; $J_{j}$ is the component diffusion flux, and $S_{h}$ is the volume heat source due to the heat transfer between droplet and the continuous phase.

The standard k−ε model is used for the turbulence model based on the turbulent flow energy and the diffusivity. The turbulent kinetic energy equation is an exact equation while the diffusivity equation is derived from empirical formulas. The turbulent kinetic energy k-equation and the diffusivity ε-equation are described as Equations (4) and (5) respectively [2]:

(4) $$\rho \frac{Dk}{Dt}=\frac{\partial }{\partial x_{i}}\left[\left(\mu_{l}+\frac{\mu_{t}}{\sigma_{k}}\right)\frac{\partial k}{\partial x_{i}}\right]+G_{k}+G_{b}-\rho \varepsilon$$

(5) $$\rho \frac{D\varepsilon }{Dt}=\frac{\partial }{\partial x_{i}}\left[\left(\mu_{l}+\frac{\mu_{t}}{\sigma_{\varepsilon }}\right)\frac{\partial \varepsilon }{\partial x_{i}}\right]+C_{1\varepsilon }\frac{\varepsilon }{k}\left(G_{k}+G_{3\varepsilon }G_{b}\right)-C_{2\varepsilon }\rho \frac{\varepsilon^{2}}{k}$$

where $\mu_{l}$ and $\mu_{t}$ are the viscous coefficients of laminar and turbulence respectively; $G_{k}$ and $G_{b}$ are the turbulence energies due to the laminar velocity gradient and buoyant force respectively; $C_{\mu }$ is the turbulent constant; $C_{1\varepsilon }$, $C_{2\varepsilon }$, $C_{3\varepsilon }$, $\sigma_{k}$ and $\sigma_{\varepsilon }$ are all empirical constants whose values are 1.44, 1.92, 0.09, 1.0, and 1.3 respectively.

#### 2.2.2. Discrete Phase Model

The air-blast nozzle, which existed in the model library of the FLUENT, was used to investigate the spray characteristic. The air-blast atomization process is to accelerate the breakup of liquid sheets from the atomizer by means of an additional air stream passing through the atomizer. ANSYS FLUENT’s air-blast atomization model assumes that the sheet breakup is always due to short waves. This assumption is a consequence of the greater sheet thickness commonly found in air-blast atomizers. Hence the ligament diameter is assumed to be linearly proportional to the wavelength of the fastest-growing wave on the sheet, and which can be calculated by Equation (6).

(6) $$d_{L}=\frac{2\pi C_{L}}{K_{s}}$$

where $C_{L}$ is the ligament constant which is equal to 0.5 by default; where $K_{s}$ is the wave number corresponding to the maximum growth rate.

In the atomization field, KHRT (Kelvin–Helmholt–Rayleigh–Taylor) is used to solve the two- phase flow. The breakup process is the result of the interaction of the KH model and the RT model, and the formation liquid radius and breakup time can be calculated by Equations (7) and (8) respectively:

(7) $$r_{c}=\frac{\pi C_{RT}}{K_{RT}}$$

(8) $$\tau_{\mathrm{RT}}=\frac{C_{\tau }}{\Omega_{RT}}$$

(9) $$\Omega_{\mathrm{RT}}=\sqrt{2{\left(-g_{t}\left(\rho_{p}-\rho_{g}\right)\right)}^{\frac{3}{2}}/3\sqrt{3\sigma }\left(\rho_{p}+\rho_{g}\right)}$$

(10) $$K_{RT}=\sqrt{-g_{t}\left(\rho_{p}-\rho_{g}\right)/3\sigma }$$

where $\Omega_{\mathrm{RT}}$ is the fastest growing unstable wave frequency of the jet; $K_{RT}$ is the wave number; $\rho_{p}$ and $\rho_{g}$ are density of liquid droplet and gas-phase respectively; σ is the surface tension.

The particle motion equation in Lagrange coordinates can be obtained by Newton’s second law:

(11) $$\left[\begin{array}{l} \frac{du_{p,x}}{dt}=F_{D}\left(u_{x}-u_{p,x}\right)+\frac{g_{x}\left(\rho_{p}-\rho_{v}\right)}{\rho_{p}}+F_{x}\\ \frac{du_{p,y}}{dt}=F_{D}\left(u_{y}-u_{p,y}\right)+\frac{g_{y}\left(\rho_{p}-\rho_{v}\right)}{\rho_{p}}+F_{y}\\ \frac{du_{p,z}}{dt}=F_{D}\left(u_{z}-u_{p,z}\right)+\frac{g_{z}\left(\rho_{p}-\rho_{v}\right)}{\rho_{p}}+F_{z} \end{array}\right]$$

where u is the velocity of the liquid-phase, m/s; $u_{p}$ is the velocity of the liquid droplet particle, m/s; $F_{D}$ is the drag force on the droplet of unit mass, m/s^2^.

#### 2.2.3. Coupling Calculation of Continuous Phase and Discrete Phase

The numerical investigation of spray in ANSYS FLUENT belongs to a two-phase simulation of air and liquid coupling calculation. As a continuous phase, air has an effect on the orbital distribution as well as the heat and mass transfer of droplets in the discrete term; in turn, the discrete phase will also affect the continuous phase. The change values of momentum, mass and heat of droplets, which are achieved by the source term, can be calculated by Equations (12) to (14):

(12) [Fx=(3μCDRe4ρddp4(uv,x−up,x)+gx(ρp−ρv)ρp−DT,P1mpT∂T∂x)mp′⋅ΔtFy=(3μCDRe4ρddp4(uv,y−up,y)+gy(ρp−ρv)ρp−DT,P1mpT∂T∂y)mp′⋅ΔtFz=(3μCDRe4ρddp4(uv,z−up,z)+gz(ρp−ρv)ρp−DT,P1mpT∂T∂z)mp′⋅Δt]

(13) $$M=\frac{\Delta m_{p}}{m_{p,0}}m_{p,0}^{\prime }$$

(14) $$Q=\left(\frac{\bar{m_{p}}}{m_{p,0}}c_{p}\Delta T_{p}+\frac{\Delta m_{p}}{m_{p,0}}\left(-h_{fg}+\int_{T_{ref}}^{T_{p}}c_{p,h_{2}o}dT\right)\right)m_{p,0}^{\prime }$$

### 2.3. Boundary Conditions and Coupling Calculation Procedure

Reasonable setting of the boundary conditions of the computational model according to the physical environment of the experiment can not only accelerate the convergence speed of the simulation process, but also improve the accuracy of the calculation. The present study set the corresponding initial state and boundary conditions according to the actual working conditions of the spray experiment, specifically as follows:

(1) The pressure, temperature, and gravity acceleration of the initial states were 1 atm, 293 K, and 9.8 m/s^2^ respectively. The injection direction was Y-axis;
(2) The velocity of mass-flow-inlet of central-flow, velocity-inlet of swirling-flow and the pressure-inlet were 9.167 × 10^−5^ kg/s, 20 m/s, and 1m/s respectively, whose values were all obtained from the experimental data. The rotating tangential vector of the swirling-flow was 0.7071 and the gas-phase was air;
(3) The vertical wall surface was defined as an escape and no-slip boundary condition and the bottom side was the pressure outlet;
(4) The target surface was no-slip condition and the formation of liquid film due to atomized droplets impinging on the surface was simulated by the FLUENT’s Eulerian-wall film model.

**Figure 2:**
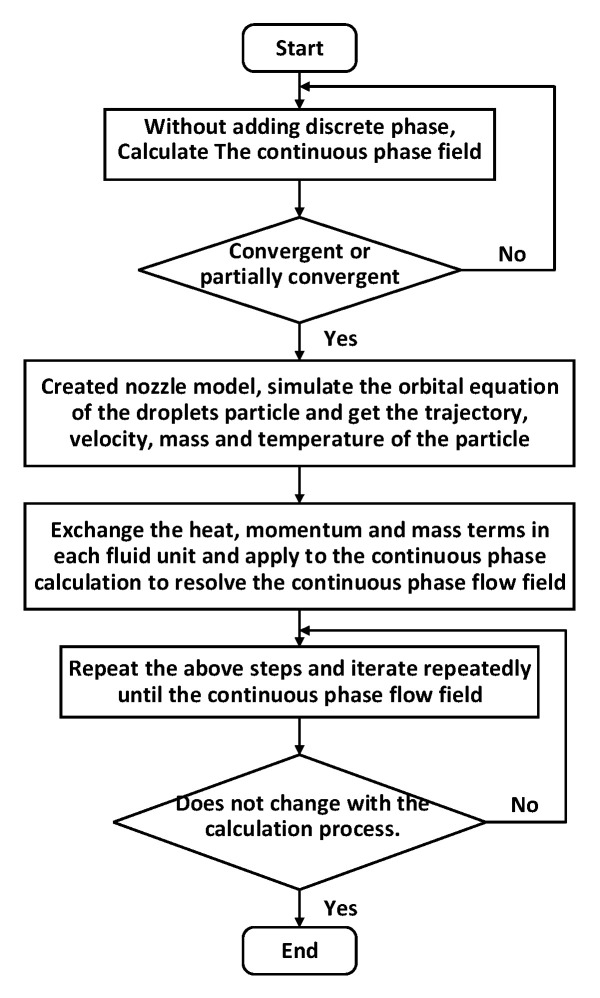
Flowchart of the calculation procedure.

The present study assumed that the thickness of liquid film was less than 500 μm and the simulation process was transient with an unsteady solution method. The coupled calculation of continuous phase and discrete phase could be carried out in the following step, which is shown in Figure 2 and described as follows: (1) Without adding the discrete phase, calculate the convergent or partially convergent continuous phase field; (2) Create a nozzle model for adding the discrete phase to simulate the orbital equation of the droplet particles and obtain the trajectory, velocity, mass and temperature of the particle; (3) Exchange the heat, momentum, and mass terms in each fluid unit and apply to the continuous phase calculation to resolve the continuous phase flow field; (4) Repeat the above steps and iterate repeatedly until the continuous phase flow field does not change with the calculation process.

### 2.4. Simulating Condition Arrangement

The target of the current investigation is to study the effects of SH and SMFR on the thermodynamic characteristics of the liquid film as well as the average droplets SMD, which all influence the heat transfer performance of the spray cooling technology using the air-blast nozzle. The related simulating operation conditions are listed in Table 1. Transparently, cases 1 to 4 were arranged to study the influence of various SHs, while the cases 2, 5–7 were arranged to research the effect of SMFRs on the thermodynamic characteristics of the liquid film and spray droplet size. It should be noted that the heat flux was 100 W/cm^2^ in the present study.

## 3. Results and Discussion

**Figure 3:**
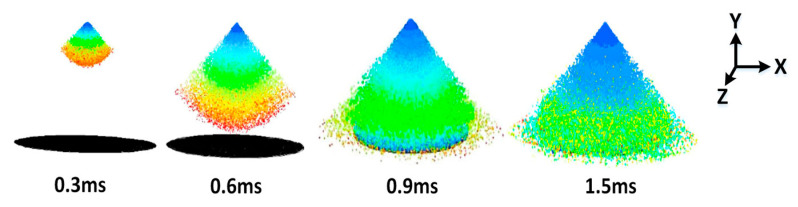
Cloud pictures of spray process.

The thermodynamic characteristics of the air-blast atomization spray cooling technology were simulated with an explored 3-D model using the commercial software of ANSYS 15.0 (ICEM CFD and FLUENT), exploring the effects of SH (15 mm, 20 mm, 25 mm, and 30 mm) and SMFR (0.020 kg/s, 0.025 kg/s,0.030 kg/s, and 0.035 kg/s) on SMD of the droplet and the thermodynamic characteristics of the liquid film (LFH, LFV, and LFGR). Figure 3 shows the cloud picture of the spray process under an SH of 30 mm. It took 0.6 ms for the liquid droplets from the nozzle to reach the surface and the spray completely covered the surface in 0.9 ms.

### 3.1. Effects of the SH

The distance between the nozzle outlet and the target surface has a great influence on the thermodynamic property of the generated liquid film upon the target surface. The aim in the current section is to explore the effects of SH on the air-blast atomization spray cooling process, seeking efficient thermodynamic characteristics of the obtained droplets and thin liquid film.

#### 3.1.1. SMD of the Spray Droplet Size

**Figure 4:**
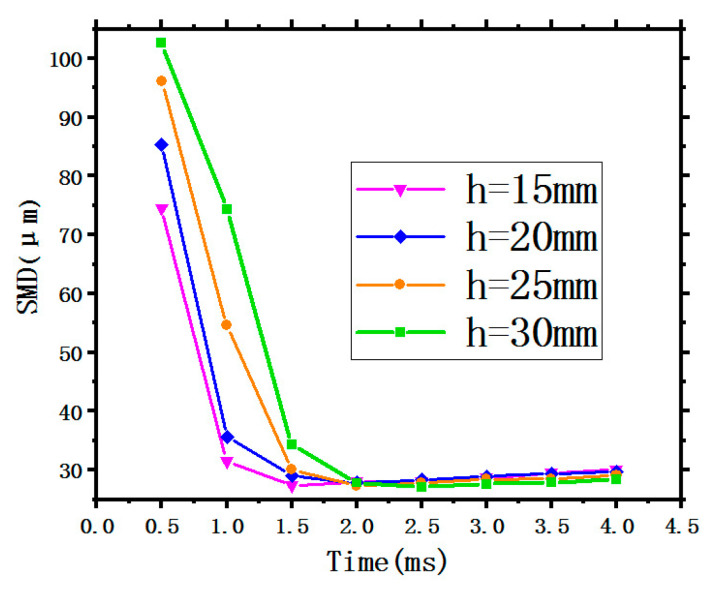
Variation of the SMD with different SH.

The SMD is a critical factor to measure the atomization quality and affect the performance of spray cooling. In general, a better spray cooling performance can be acquired by decreasing the SMD of the spray droplet size [10]. Figure 4 shows the variation of the SMD with time under different SHs of 15 mm, 20 mm, 25 mm, and 30 mm.

In Figure 4, it is obvious that the average SMD of the droplet size has a sharp drop being sprayed from the nozzle in a short period of time (<1.5 ms), then approaching smoothness where it is below a value of 40 μm where the spray status tends to be stable. The three curves show the same trend, indicating that the SH has little effect on the SMD because of the same initial conditions with a constant spray flow rate of 0.025 kg/s and three invariable inlet conditions as previously mentioned.

#### 3.1.2. The Hydraulic Characteristics of the Liquid Film

The main hydraulic characteristics of the liquid film are all discussed in detail as follows, including the liquid film height (LFH), the liquid film velocity (LFV), and the liquid film generation rate (LFGR) under different SH. Especially, the cloud pictures and the corresponding curves were all obtained under the condition in which the liquid film completely covered the target surface with a time of 3.5 ms.

1. The LFH and the LFV

**Figure 5:**
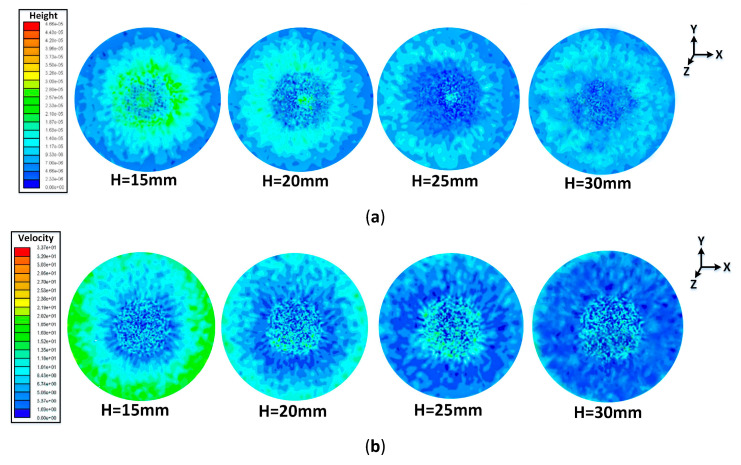
Cloud pictures of the liquid film: (**a**) the LFH and (**b**) the LFV.

The cloud pictures including the LFH and LFV are displayed in Figure 5a,b respectively. It is obvious that the variation of the LFH and the LFV present a circular distribution with the center below the nozzle. Near the central area, the atomization droplets directly impinged on the surface and were just below the jet center. With the rebound and splash of a great amount of droplets, the LFH in this area is low and the LFV has a large speed fluctuation. After the liquid film spreads rapidly along the radius, the LFH presents a trend of first increasing and then decreasing, while the LFV shows the opposite trend. This is due to the extrusion and flow of a large number of droplets around the central area, resulting in the growth of the LFH and the LFV. On continuing outward, the liquid film obtains an adequate diffusion space so that the LFV continues to increase, while the LFH gradually decreases due to the momentum conservation law. In addition, comparing with the LFH under the four SHs (15 mm, 20 mm, 25 mm, and 30 mm), it is found that the average LFH at the nozzle height of 15 mm is higher than that of the other three heights as well as the LFV being the largest. However, the distribution of liquid film under the highest SH condition of 30 mm is the most uniform on the whole target surface.

**Figure 6:**
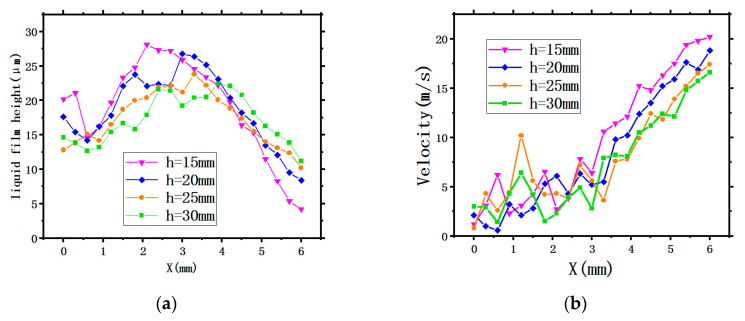
The thermodynamic characteristic curves of the liquid film: (**a**) the LFH and (**b**) the LFV.

For the purpose of better studying the flow characteristics of the liquid film, several points along the radius of the X-axis on the circular surface were taken to quantitatively analyze the LFH and the LFV under different SH conditions. The specific hydraulic characteristic curves distribution is shown in Figure 6. The maximum LFHs of 28.1 μm, 26.8 μm, 23.8 μm, and 22.4 μm appear at X = 2.1 mm, X = 3 mm, X = 3.3 mm, and X = 3.9 mm respectively. The lower SH has a higher maximum LFH due to the smaller projection circle of the spray on the surface, resulting in a reduction in the radius of the central area directly affected by the droplets of the atomized main flow region. It can be seen from Figure 6. Figure 6b that at the area near the center, the LFV fluctuates greatly under different SH conditions especially for a nozzle height of 30 mm. After leaving the central area, the LFV shows an increasing trend, and the lower the SH, the larger the LFV. The reason being the LFV is affected by the droplet impact, splash, and collision in the central area. With the growing amount of spray, the liquid film flows rapidly around and the LFV is gradually increased, reaching a maximum value at the edge of the target surface.

2. The LFGR

When the atomization droplets impinge on the target surface, a liquid film is formed, whose quality is also a critical factor influencing the ability of the spray cooling technology. The film generation rate (∂) is used for comparing the ratio of the atomization droplets impinging and forming liquid film on the surface ($m_{lf}$) to total amount of the working fluid (m) under different conditions. The calculation can be expressed by Equation (15) [18].

(15) $$\partial =\frac{m_{lf}}{m}$$

**Figure 7:**
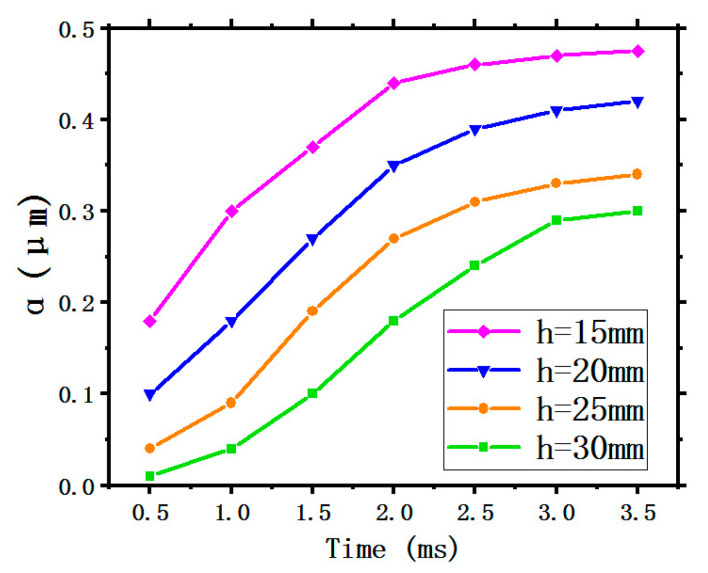
The film generation rate.

Figure 7 shows the variation curves of the LFGR with time under different SHs. It is obvious that all four curves show an upward trend first then gradually flatten, indicating that the formation of liquid film gradually completes to an invariable shape meaning that the LFH and the LFV are fixed in a range. The LFGR under an SH of 15 mm is higher than that of the other three SHs, because the decrease of SH narrows down the atomization range and less droplets collide and splash during the movement, resulting in the supplementation of the quality of liquid film formation as well as an increase of the LFGR.

### 3.2. Effects of the SMFR

The SMFR of the input coolant that are charged into the air-blast nozzle will have a major effect on the property of the thermodynamic characteristics. In this section, four different SMFRs of 0.020 kg/s, 0.025 kg/s, 0.030 kg/s, and 0.035 kg/s are changed to investigate the influence on the thermodynamic properties of the generated droplets and the characteristics of the liquid film on the target surface.

#### 3.2.1. SMD of the Spray Droplet Size

**Figure 8:**
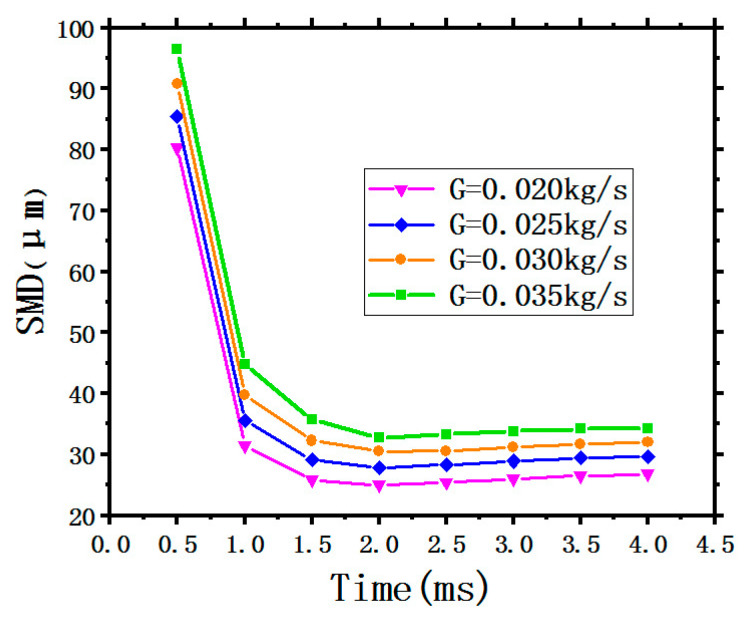
Variation of the average droplets SMD with different SMFRs.

Figure 8 shows the variation of the average droplets SMD with time under different SMFRs. It can be observed obviously that all four curves show similar trends, the average SMD first has a sharp drop with a short period time of 1.5 ms, then approaching smoothness below the value of 40 μm. With the growth of SMFRs from 0.020 kg/s to 0.035 kg/s, the relatively positions of the four curves increase. The green curve representing the maximum SMFR of 0.035 kg/s stays at the highest position, indicating that the average droplets SMD generated by the maximum SMFR have the largest value.

Generally speaking, liquid coolant comes into the nozzle, where it is mixed with high-pressure air then atomized into thin liquid droplets. The SMD of the generated droplets depends on the state of the nozzle inlet including SMFR and the high-pressure air. Therefore, the average droplet SMD increases with increasing SMFR under an invariable air-inlet-pressure.

#### 3.2.2. The Hydraulic Characteristics of the Liquid Film

1. The LFH and the LFV

**Figure 9:**
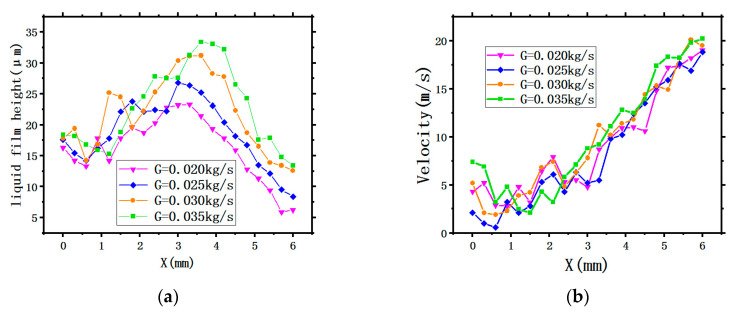
The thermodynamic characteristic curves of the liquid film: (**a**) the LFH and (**b**) the LFV.

Figure 9 shows the thermodynamic characteristic curves of the liquid film. Several points along the radius of the X-axis on the circular surface were taken to quantitatively analyze the LFH and LFV under different SMFRs.

Figure 9a shows the characteristic curves of the LFH. All four curves representing four different SMFRs display the similar tendency. It can be clearly observed that all LFH generated by the four different SMFRs are all under the value of 35 μm. Within a radius of about 3 mm, the SMFR has little effect on the LFH; while the liquid film gradually expands outward along the radius (>3 mm), the average LFH will increase with the growth of SMFR. Additionally, the peak value of LFH also increases with increasing SMFR.

Figure 9b displays the characteristic curves of the LFV. The tendency of the curves is similar to that generated by the different SHs in Section 3.1.2. However, the four curves representing the four SMFRs also have similar values, meaning that the LFV with different SMFRs have similar velocity distribution, indicating that the SMFR has little effect on the LFV.

2. The LFGR

**Figure 10:**
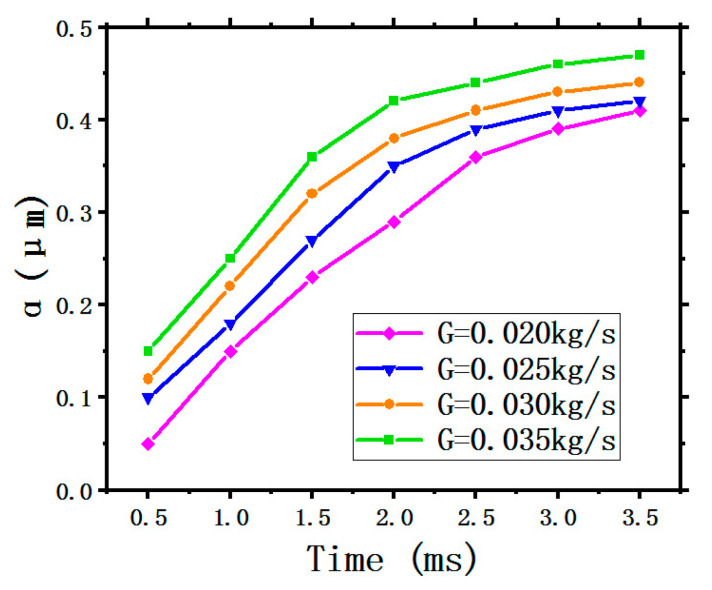
The film generation rate.

Figure 10 illustrates the curves of the LFGR. All four curves display the same tendency as time goes on under the different SMFRs, where all the four curves show an upward trend first then gradually flatten. This indicates that the formation of liquid film gradually completes to an invariable shape meaning that the LFH and the LFV are fixed in a range. The green curve stays at the highest position, indicating that the LFGR generated by the maximum SMFR of 0.035 kg/s has the highest ability. A greater SMFR means more coolant spray onto the target surface, thus the LFGR will increase with increasing SMFR. In addition, the LFGRs obtained by the four different SMFR are all lower than 0.5.

## 4. Conclusions

In the present paper, a three-dimensional model was developed to investigate both the thermodynamic characteristics of a liquid film with an air-blast nozzle for spray cooling and the spray quality. The model focuses on simulating the factors including the SMD of spray droplet size, the LFH, the LFV, and the LFGR, which all influence the heat transfer performance of spray cooling. The variation of factors under different SHs and SMFRs are discussed in detail. The primary conclusions can be formulated as follows:

(1) The varied SH has little effect on the SMD with an invariable inlet condition; while the different SMFRs can influence the SMD of the spray droplet. With increasing SMFR, the SMD becomes larger. The average SMD of droplet size shows a sharp drop when sprayed from the nozzle in a short period of time (<1.5 ms), then approaching smoothness below a value of 40 μm, the spray status tends to be stable;
(2) Under all working conditions, the variations of LFH and LFV have a similar tendency. In the central area below the nozzle on the surface, the LFH is relatively low as well as the LFV while the LFV has a large speed fluctuation. Along the radius direction, the LFH increases first and then decreases, while the LFV increases gradually. When it approaches the circumferential edge, the velocity reaches its maximum;
(3) A larger LFGR can be obtained by a lower SH. With the increase of SH, the average LFH decreases as well as the peak height but is higher at the edge-area. The LFV expands from the central region to the surrounding areas. A lower SH can be obtained a a higher average LFV and a lower speed fluctuation in the central area.
(4) With the growth of SMFR, the LFGR and the average LFH become larger. In addition, the peak value of LFH also increases with increasing SMFR. The LFVs with different SMFRs have similar velocity distribution, indicating that the SMFR has little effect on the LFV.

Overall, the SH and the SMFR have major effects on the thermodynamic characteristics of the liquid film as well as the spray quality, which would have a large influence on the heat transfer performance of air-blast spray cooling technology. From the above results and conclusions, we find a promising approach to achieve a more efficient heat dissipation capacity with spray cooling technology.

## Figures and Tables

**Table 1:** Description of simulating conditions.

Case	Various Parameter Descriptions	
	SH (mm)	SMFR (kg/s)
1	15	0.025
2	20	0.025
3	25	0.025
4	30	0.025
5	20	0.020
6	20	0.030
7	20	0.035

## Nomenclature

C	constant	$\tau_{\mathrm{RT}}$	breakup time
D	Diameter (m)	iε	Empirical (i = 1,2 and 3)
$d_{l}$	ligament diameter	**Subscripts**	
$d_{p}$	Particle diameter	b	buoyant
E	specific energy including kinetic energy, intrinsic energy and potential energy	D	drag
$F_{D}$	drag force on droplet of unit mass (m/s^2^)	g	gas-phase
$G_{b}$	turbulence energy due to the buoyant force	L	ligament
$G_{k}$	turbulence energy due to the laminar velocity gradient	l	laminar
g	Gravitational acceleration (m/s^2^)	p	liquid droplet particle
$J_{j}$	component diffusion flux	RT	Rayleigh–Taylor model
$K_{s}$	wave number corresponding to the maximum growth rate	t	turbulence
$K_{RT}$	wave number	x	x-axis
Q	Heat (W)	y	y-axis
Re	Reynold number	z	z-axis
$r_{c}$	liquid radius	**Acronyms**	
$S_{h}$	volume heat source due to the heat transfer between droplet and the continuous phase	CFD	Computational Fluid Dynamics
T	Temperature (K)	DPM	Discrete Phase Model
u	velocity of liquid-phase (m/s)	LFH	Liquid Film Height
**Greek symbols**		LFV	Liquid Film Velocity
λ	Thermal conductivity ($W\cdot m^{-1}\cdot K^{-1}$)	LFGR	Liquid Film Generation Rate
ζ	Uncertainty (%)	LD-MOSFET	Lateral Diffused Metal Oxide Semiconductor Field Effect Transistor
ρ	Density ($kg\cdot m^{-3}$)	SH	Spray Height
σ	Surface tension ($N\cdot m^{-1}$)	SMD	Sauter Mean Diameter
Ω	unstable wave frequency	SMFR	Spray Mass Flow Rate
μ	Dynamic viscosity (Pa⋅s)	3-D	Three dimensional
